# c-Cbl: An Important Regulator and a Target in Angiogenesis and Tumorigenesis

**DOI:** 10.3390/cells8050498

**Published:** 2019-05-23

**Authors:** Chimera L. Lyle, Mostafa Belghasem, Vipul C. Chitalia

**Affiliations:** 1 Department of Medicine, Boston University Medical Center, Boston, MA 02118, USA; clyle@bu.edu; 2 Department of Pathology and Laboratory Medicine, Boston University Medical Center, Boston, MA 02118, USA; mbelgha@bu.edu; 3 Boston Veterans Affairs Healthcare System, Boston, MA 02118, USA

**Keywords:** angiogenesis, tumors, ubiquitination, proteasomal degradation, c-Cbl, RTK, non-RTK, VEGFR, FGFR, PDGFR, EGFR, c-Met, Wnt signaling, β–catenin, PLCγ1

## Abstract

Casitas B lineage lymphoma (c-Cbl) is a multifunctional protein with a ubiquitin E3 ligase activity capable of degrading diverse sets of proteins. Although previous work had focused mainly on c-Cbl mutations in humans with hematological malignancies, recent emerging evidence suggests a critical role of c-Cbl in angiogenesis and human solid organ tumors. The combination of its unique structure, modular function, and ability to channelize cues from a rich network of signaling cascades, empowers c-Cbl to assume a central role in these disease models. This review consolidates the structural and functional insights based on recent studies that highlight c-Cbl as a target with tantalizing therapeutic potential in various models of angiogenesis and tumorigenesis.

## 1. Introduction

The formation of new blood vessels is fundamental to health and disease[1,2,3,4,5]. This formation can occur by two processes: vasculogenesis or angiogenesis. In vasculogenesis, new vessels are created from angioblasts or other precursor cell, which then differentiate into endothelial cells forming lumens and primordial blood vessels. In contrast, in angiogenesis preexisting vasculature is the source of new capillary formation. Angiogenesis is a dynamic process orchestrated by endothelial cells (ECs), their associated perivascular supporting cells (vascular smooth muscle cells and pericytes), and immune cells. Dysregulation of angiogenesis has been recognized as a commonality in a myriad of pathological disorders including cancer [6], peripheral artery disease (PAD) [7], diabetic retinopathy [8], rheumatoid arthritis [9], and inflammatory bowel disease (IBD), etc. [10].

Angiogenesis is regulated by a delicate and dynamic balance of growth factors, intracellular signaling pathways, and extracellular components. Several mediators are involved in this process, including basic fibroblast growth factor (BFGF) [11,12], transforming growth factor (TGF)-α [13], TGF-β [14,15], tumor necrosis factor (TNF)-a [16,17], vascular endothelial growth factor VEGF [2,18,19,20], β-catenin [21,22,23], and Phospholipase C gamma (PLCγ1) [24,25,26,27]. The growth factors secreted from a medley of different cell types including endothelial cells, smooth muscle cells, fibroblasts, platelets, and immune cells coordinate this process. Physiologic and pathologic stimuli such as chronic inflammation, injury, hypoxia, and cancer further modulate specific growth factors within the extracellular milieu to influence the angiogenesis. This review specifically describes the role of Casitas B lineage lymphoma (c-Cbl) in different models of angiogenesis.

## 2. c-Cbl Family of Ubiquitin E3 Ligases

Casitas B-lineage lymphoma (c-Cbl) is a member of the CBL family of proteins and is comprised of three homologues [28,29]. c-Cbl is an ubiquitously expressed mammalian gene that plays a vital role in fundamental cellular functions including cell survival, migration and proliferation. v-Cbl (viral casita B-lineage lymphoma), the first member of the Cbl family, was discovered in 1989 [30]. It is a 357 amino acid protein from the Cas NS-1 murine retrovirus, which was found to induce pre-B cell lymphomas and myelogenous leukemia in mice obtained from the Lake Casitas, California [30]. Further studies showed that CAS NS-1 originated from the ecotropic (a virus that can only infect mouse or rat cells) CAS-Br-M virus resulting from continuous recombination with a cellular oncogene and endogenous retroviral sequences [31]. The truncated virally encoded protein mutant was named v-Cbl to distinguish it from normal mouse c-Cbl (906–973 amino acid), which failed to induce the formation of tumors [28,30,31,32]. Another homologue of c-Cbl is Cbl-b, which is expressed in normal and malignant mammary epithelial cells, hematopoietic cell lines/tissues, as well as various normal tissue lines [33,34,35,36].

**Figure 1:**
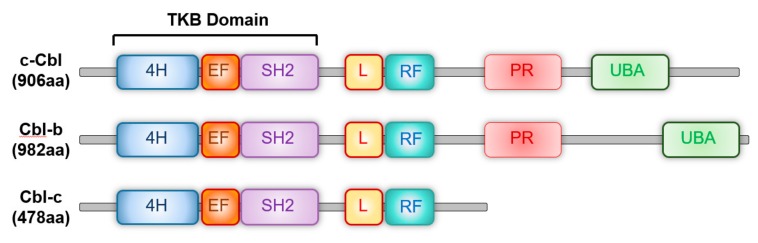
The primary structure and domain organization of human Casitas b-lineage lymphoma family proteins. c-Cbl, Cbl-b and Cbl-c proteins contain highly conserved tyrosine kinase-binding (TKB), linker (L), RING finger (RF) and proline-rich (PR) domains. 4H: Four-helix bundle; EF: EF hand, SH2: Src-homology 2; UBA: Ubiquitin-associated domain.

Structurally, c-Cbl has several regions that encode functionally distinct protein domains (Figure 1). An N-terminal tyrosine kinase binding (TKB) domain consisting of four helix bundles, an EF hand and a SH2 domain. It binds specifically to the receptor and non-receptor tyrosine kinases. The RING finger domain is the E3 ubiquitin ligase and mediates ubiquitination of its targets. The C terminus has a proline rich domain that represents the protein-protein interaction domain and mediates the interaction of c-Cbl with various targets. Three exposed tyrosines in this region Tyr700, Tyr731 and Tyr744, modulate the interaction of c-Cbl with downstream signaling molecules such as Akt and other proteins. The C-terminal ubiquitin associated domain (UAB) functions as the site of ubiquitin binding and also facilitates the dimerization of c-Cbl [22]. v-Cbl retains the TKB domain, but lacks the RING finger and the C-terminal domains. Studies have shown that a deletion mutation in a region close to the RING finger results in c-Cbl becoming oncogenic [37] . Cbl-b is a larger protein than c-Cbl and contains an additional 69 amino acids at the C-terminus. 70Z-c-Cbl is a naturally occurring mutation lacking a 17-amino acids region within the linker domain upstream of the RING finger. It lacks the E3 ligase activity and serves as a dominant negative for several of c-Cbl’s targets [21,38].

In addition to the adaptor function of c-Cbl, activity of c-Cbl in angiogenesis is in large part driven by its E3 ubiquitin ligase function [39,40]. Ubiquitination is a critical post translational protein modification (PTM) [41]. It regulates fundamental cellular processes and is characterized by the covalent binding of the C-terminal glycine located on the ubiquitin molecule to a lysine residue on a substrate protein. Polyubiquitinated proteins are targeted for lysosomal or proteasomal degradation [42,43,44,45]. This specificity to the ubiquitination process is imparted by E3 ubiquitin ligase that recognize the substrate and also interacts and transfers E2 ubiquitin to the substrate.

The bulk of c-Cbl’s cellular effects are linked to its RING finger and E3 ubiquitin function. Studies have shown that the ubiquitin activity of c-Cbl is regulated by Tyr371, a key tyrosine residue. Recent X-ray crystal structural and point mutagenesis studies have provided interesting insight into c-Cbl structure and functional analysis [28,46]. Dou et al solved the crystal structures of human c-CBL; a c-CBL−substrate peptide complex and a phosphorylated-Tyr371-CBL−E2−substrate peptide complex and compared them with the known structure of a c-CBL−E2−substrate peptide complex [29]. Structural and biochemical analyses showed that c-CBL adopted an autoinhibited RING conformation such that the binding surface of the RING finger exhibited reduced affinity for E2. c-Cbl ubiquitination activity was induced by phosphorylation of a linker helix region (LHR) Tyr371. Tyr371 phosphorylation induced a conformational change in the LHR domain to eliminate autoinhibition and flipped the RING domain to convert the RING domain into an enhanced E2-binding module. These results demonstrated the importance of Tyr371’s phosphorylation in regulating c-Cbl’s activity.

c-Cbl is a RING finger E3 ubiquitin ligase and targets several pro-angiogenic mediator proteins for degradation including receptor and non-receptor tyrosine kinases (c-Src, c-MET, EGFR, PDGFR, etc), mediators of Wnt signaling (β-catenin) [21,22,47,48], and VEGF signaling (PLCγ1) [25,38]. Thus, c-Cbl is able to both modulate and regulate angiogenesis. The significance of c-Cbl is underscored by the fact that endothelial cells from c-Cbl knockout mice exhibited increased cell proliferation and tube formation following stimulation with VEGF. Mice with a c-Cbl knockout showed increased evidence of physiologic angiogenesis in several models such as chorioalloantoic membrane angiogenesis [25,38], zebrafish model [22], pathologic angiogenesis in tumor-induced angiogenesis [25], and retinal neovascularization [24].

## 3. c-Cbl as a Major Regulator of Angiogenesis

### 3.1. c-Cbl and β-Catenin

Wingless/Integrated (Wnt) signaling is a critical mediator of angiogenesis [49,50,51]. The canonical as well as the noncanonical Wnt signaling pathways are activated by engagement of Wnt ligands to their cogent receptors SFRP and Frizzled. The central mediator of canonical Wnt signaling is β-catenin, which translocates into the nucleus of endothelial cells and induces transcription of several pro-angiogenetic mediators such as VEGF-A and IL-8 [50]. In contrast, the noncanonical Wnt signaling is β-catenin independent and is critical for both the transcriptional and non-transcriptional cellular responses [52]. For example, the planar cell polarity (PCP) pathway, a non-canonical Wnt pathway, regulates cell polarity by controlling gene expression and rearrangements in the cytoskeleton via the effects of c-Jun N-terminal kinase (JNK) and Ras homologue gene family, member A [53]. The Wnt-Ca^2+^ pathway is mediated through the hetertrimeric G proteins [54] These proteins activate phospholipase C (PLC) resulting in intracellular storage Ca^2+^ release and transcription of genes responsible for cell migration and cell fate [54].

The canonical Wnt pathway is inhibited by noncanonical Wnt signaling [55,56]. β-catenin–TCF dependent transcription has been shown to be inhibited through TCD phosphorylation via CaMKII-TAK1-NLK pathway activation. Similarly, NFAT has been shown to suppress β-catenin transcription [55,57]. While several angiogenic disorders are linked to mutations in the canonical Wnt signaling pathway [49,51,58,59,60,61], disorders involving cellular polarity and motility result from disruptions in the noncanonical Wnt signaling pathway.

**Figure 2:**
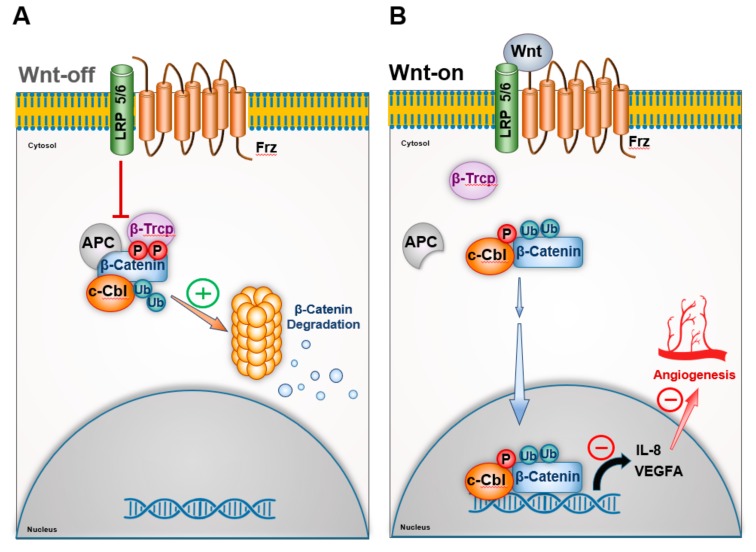
c-Cbl regulates angiogenesis through Wnt signaling. Constitutively Wnt signaling is suppressed in endothelial cells. In absence of Wnt ligand, β-Catenin is downregulated by in a destruction complex in the cytosol consisting of Adenomatous Polyposis Coli (APC), where β-Catenin gets phosphorylated by GSK3β. This results in β-catenin ubiquitination and proteasomal degradation by E3 ligases such as c-Cbl and β-TrCP, etc. Wnt ligand activates Frz receptors, which dissociates the destruction complex, β-catenin undergoes nuclear translocation and activates the Wnt target genes such as IL-8 and VEGF-A to induce angiogenesis. Wnt activation results in phosphorylation of c-Cbl, its nuclear translocation, wherein it ubiquitinates nuclear β-catenin to switch-off the Wnt signaling and angiogenesis.

Under normal circumstances, canonical Wnt signaling is constitutively inhibited by the degradation of β–catenin (Wnt-off phase) [58]. Binding of the Wnt ligand to its cogent receptor stabilizes β–catenin, which undergoes nuclear translocation (Wnt-on phase). The pro-angiogenic function of Wnt signaling is largely driven by nuclear β–catenin in Wnt-on phase. Work of Chitalia et al demonstrated that c-Cbl directly interacted and suppressed β–catenin in both Wnt-off and Wnt-on phase in the endothelial cells in different subcellular compartments [21] (Figure 2).

The site of interaction of c-Cbl and β–catenin imparted this functional modularity to c-Cbl. The C-terminus of c-Cbl interacts with the central region of β–catenin containing Armadillo repeat region and targets the cytosolic β –catenin to proteasomal degradation in Wnt-off phase [21]. In Wnt-on phase, c-Cbl undergoes nuclear translocation in a manner similar to that of β–catenin, degrades nuclear β–catenin, and suppresses transcription induction of pro-angigoenic VEGF-A and IL-8 cytokines. Shivanna et al. further showed that activation of the Wnt signaling pathway promoted the phosphorylation of c-Cbl at tyrosine 731(Tyr-731) [22].

Phosphorylation of Tyr731 increased its dimerization through its UBA domain as well as the nuclear translocation of c-Cbl and also increased its interaction with the nuclear β–catenin. This event augmented the degradation of nuclear active β–catenin in the Wnt-on phase and suppressed Wnt signaling and angiogenesis. c-Cbl silenced cells exhibited augmented Wnt activity in endothelial cells and increased nuclear β–catenin, increased tube formation in an in vitro angiogenesis assay [21]. 

In line with the crystal structure of c-Cbl, its regulation of nuclear β–catenin requires phosphorylation of Tyr371 [48]. Inactivating mutations of Tyr371 compromised the ability of c-Cbl to target nuclear β–catenin. The c-Cbl tyrosine 371 (Y371H) mutant interacts with but fails to ubiquitinate nuclear β–catenin. The nuclear localization of the c-Cbl TyrY371H mutant contributes to its dominant negative effect on nuclear β–catenin. A transgenic Wnt-8 zebrafish model showed c-Cbl Y371H mutant augmented Wnt/β–catenin signaling, increased Wnt target genes and angiogenesis [48]. Taken together, this body of work demonstrated that c-Cbl regulated angiogenesis through Wnt signaling.

### 3.2. c-Cbl and VEGF Signaling

VEGF signaling is critical for angiogenesis as well as vasculogenesis. VEGF-A stimulates VEGF receptors 1 and 2 (VEGFR-1 and VEGFR-2) on endothelial cells to initiate a diverseness of biological responses that serve to increase endothelial cell survival, migration, and differentiation [18,19,20,62,63]. The binding of VEGF to its receptor VEGFR-2 activates another pivotal mediator of angiogenesis: PLCγ1. PLCγ1 plays an important role in mediating cellular signaling, cell migration, and actin cytoskeleton remodeling. The importance of PLCγ1 in angiogenesis was proven by the targeted deletion of PLCγ1, which resulted in early embryonic lethality between E (embryonic day) 9.5 and E10.5 due to significantly impaired vasculogenesis and erythrogenesis. Inactivation of PLCγ1 in zebrafish is also shown to be required for the function of VEGF and arterial development [22,64,65]. Pharmacological manipulation of PLCγ1 by compound U73122 inhibited endothelial cell tube formation in vitro [25,66] and angiogenesis in vivo in CAM assay [24]. In fact, activating mutations of PLCγ1 are detected in angiosarcoma that may regulate the angiogenic phenotype of the tumors [26,37,67,68].

Biochemical and functional studies have uncovered the effects of c-Cbl and PLCγ1 on VEGFR signaling [24,25,38,66,69,70]. c-Cbl interacts with PLCγ1 via its proline rich region in an non-inducible manner. However, c-Cbl interacts with VEGFR-2 via its N-terminal TKB domain with the activation of VEGF receptor signaling [38]. Specifically, the TKB domain of c-Cbl interacts with the phosphorylated VEGFR-2 at Tyr1052 and Tyr1057 [25,38]. The co-recruitment of c-Cbl and PLCγ1 to VEGFR-2 results in the formation of a complex that suppresses the tyrosine phosphorylation of PLCγ1. Although PLCγ1 undergoes ubiquitination, intriguingly it escapes proteolysis. 

Since c-Cbl suppressed VEGFR-2 signaling, loss of c-Cbl activity should augment angiogenesis. Indeed, the loss of c-Cbl was found to robustly activate PLCγ1, while increasing the release of intracellular calcium [25]. Taken together, these results demonstrated c-Cbl is a suppressor protein of angiogenesis via modulation of PLCγ1 activation and VEGF signaling.

### 3.3. c-Cbl Regulation of VEGFR-2 through Epsin

The epsins are a family of highly conserved endocytic adaptor proteins that function in the assembly of clathrin-coated vesicles and thus play important physiological roles in cellular function, such as recognizing or binding to ubiquitinated cargo and functioning as coordinators of endocytosis by interacting with the elements of the endocytic machinery (AP2 and clathrin) [71]. The epsin protein consists of an epsin NH(2)-terminal homology (ENTH) domain (responsible for promoting phospholipid interaction), two clathrin binding sequences, multiple AP2 binding sites, and multiple EPS15 homology (EH) domain binding motifs. Epsin also contains multiple ubiquitin interacting motifs (UIMs) that bind ubiquitin moiety [72].

Epsins regulate VEGFR-2 via their ubiquitin interacting motif (UIM) and augment its degradation thereby attenuating VEGFR-2 signaling [73,74]. This degradation is accomplished through c-Cbl. Song et al. demonstrated that the binding of Epsin via its UIM to the ubiquitin moieties on activated VEGFR-2 elicited the ubiquitination of epsin by c-Cbl. This process resulted in polyubiquitination and proteasomal degradation of activated VEGFR-2 by c-Cbl [73].

### 3.4. c-Cbl and Other Receptor Tyrosine Kinases

c-Cbl regulates several tyrosine kinases including Tie-2, Eph and c-Src. The following section covers those tyrosine kinase receptors that play an important role in angiogenesis and are regulated by c-Cbl.

#### 3.4.1. c-Cbl and Angiopoietin(1/2)-Tie2 Axis

The Tie2 receptor is a transmembrane tyrosine kinase highly expressed on the surface of endothelium. The ligands of Tie2 are Angiopoietin 1 and 2 and exhibit opposite functions to each other, such that their relative expression in the local milieu determine the activity of Angiopoietin(1/2)-Tie2 axis [75]. This signaling axis is considered to be an important regulator of endothelial permeability and angiogenesis [5,75,76,77]. The current model suggests that in the quiescent vasculature, Tie2 is phosphorylated at tyrosine residues in its intracellular domain and remains constitutively active. Activated Tie2 promotes the barrier function and anti-inflammation. In inflammation, Angiopoietin 2 is upregulated in endothelial cells and released from platelets to antagonize Angiopoietin-1. This event results in the inhibition of Tie2.

The role of both Tie2 and Angiopoietin-1 in developmental angiogenesis became evident when mice knockouts for the genes died in utero from defects in cardiac development and blood vessel formation [75,78,79]. Tie2 as well as Angiopoietin-1 are expressed in normal human arteries and veins [80]. Further studies demonstrated that Angiopoietin-1 but not Angiopoietin-2, was chemotactic for endothelial cells and augmented endothelial cell migration for angiogenesis. On the other hand, Angiopoietin-2 inhibited migration of endothelial cells toward angiopoietin-1.

c-Cbl downregulates Angiopoietin(1/2)-Tie2 axis and interacts with Tie-2 in a manner dependent on Angiopoietin-1 stimulation [81]. This interaction results in the internalization of Angiopoietin and polyubiquitination of Tie-2 [81]. While inhibition of Angiopoietin (1/2)-Tie2 axis by c-Cbl is likely to suppress angiogenesis, further work is needed to elucidate the importance of Angiopoietin(1/2)-Tie2 axis in pathologic angiogenesis models.

#### 3.4.2. c-Cbl and Eph Receptors

Along with ephrin ligands, Eph receptors play a key role in a variety of processes during embryonic development. Mice lacking Ephrin-B2 and double mutants deficient in EphB2 and EphB3 receptor signaling die in utero before embryonic day 11.5 (E11.5) due to defects in the remodeling of the embryonic vascular system. These mice show defects in the boundaries between arterial and venous domains [82]. These findings were consistently observed in other models, which showed that Ephrin-B2 regulated angiogenesis and lymphaniogenesis through VEGFR-3 signaling [83].

On the other hand, EphA2 regulation of angiogenesis is dependent on VEGFR-2 signaling [84]. In a retinal neovascularization model, EphrinA1 stimulated retinal microvascular endothelial cell proliferation, migration, tube formation and Ca2+ flux, and angiogenesis in rat aortic rings even in the milieu of VEGFR-2 inhibition. EphA2 is a target of c-Cbl [83,85]. The negative regulation of EphA2 mediated by c-Cbl was dependent on the activity of EphA2, as c-Cbl failed to downregulate the kinase inactive mutant of EphA2. The dominant negative mutant 70Z-Cbl also failed to suppress EphA2. Taken together, these data indicate the role of c-Cbl in Eph-mediated angiogenesis.

#### 3.4.3. c-Cbl and c-Src

c-Src is a members of the Src family of non-receptor tyrosine kinases. A large body of literature indicates their integral role in tumorigenesis and several of them also enhance angiogenesis [42,86,87]. For example, Donnini et al. observed that a pyrazolo-pyrimidine-derived c-Src inhibitor suppressed vascular endothelial growth factor production and signaling, thereby reducing angiogenesis as well as the survival of squamous carcinoma cells [88]. c-Cbl polyubiquitinates c-Src [89,90].

## 4. c-Cbl in Tumor-Mediated Angiogenesis

Tumors manipulate host blood flow to ensure their growth and further exploit the blood vessels for metastasis. Thus, angiogenesis is an integral part of tumor growth [1,5,6,10,13]. Specific models of tumor angiogenesis have demonstrated the role of c-Cbl in this process. Typically, tumor cells are implanted in mice as a xenograft (on the back of mice) or an orthografts (at the site of its natural growth. For example, colon cancer cells injected in colon, etc.) are implanted in mice and the vasculature within the tumor mass serves as a metrics for tumor angiogenesis. Meyer et al subcutaneously injected syngeneic B16F mouse melanoma cells into c-CBL knockout mice. They hypothesized that compared to wild-type c-Cbl mice, the tumors of c-CBL knockout mice would exhibit higher angiogenesis [25]. In fact, results showed significantly increased tumor growth in c-Cbl knockout mice when compared to the wild type mice; concurrent with increased tumor angiogenesis. This model specifically demonstrates the role of c-Cbl in tumor angiogenesis.

## 5. c-Cbl in Myocardial Ischemia

Critical coronary artery obstruction due to atherothrombotic coronary artery disease results in myocardial ischemia or angina and remains the leading cause of death in the world [91]. Myocardial ischemia is followed by reperfusion and remodeling of the cardiac myocytes. Given the role of c-Cbl in angiogenesis, Rafiq et al investigated c-Cbl in recovery from myocardial ischemia and demonstrated that mice lacking c-Cbl showed an increased functional recovery following myocardial ischemia [92]. A significant reduction in myocardial cell apoptosis was noted in these mice which augmented cardiac function post injury [92]. Loss of c-Cbl was correlated with an enhanced expression of angiogenic factors such as VEGF and its receptor VEGFR-2, as well as increased expression of epidermal growth factor receptor (EGFR) and focal adhesion kinase (FAK) [92]. Taken together, the loss of c-Cbl was cardio protective and elicited this effect by preventing or reducing the ubiquitination and degradation of various critical angiogenic factors.

## 6. c-Cbl in Retinal Neovascularization

Choroidal neovascularization retinal angiogenesis is considered the pathognomonic feature of age-related macular degeneration [24,93,94,95]. VEGF signaling has been identified as an important contributor to this disease [95,96,97]. c-Cbl knockout mice were subjected to laser-induced *choroidal neovascularization *(CNV) formation using the laser photocoagulation method [24,25]. The mice were followed by fundus photography and angiography to document the size and leakage from the CNV in these lesions using fluorescein angiography. c-Cbl knockout (KO) status was associated with a two-fold greater number of leaking CNV lesions compared with the wild-type mice. The CNV lesions were more aggressive and a higher number of lesions displayed confluence in c-Cbl KO mice. This data strongly suggested that c-Cbl is a negative regulator of CNV. While both of the above models suggest the role of c-Cbl in a model of pathological angiogenesis, its role in other models such as hind limb ischemia remains to be investigated.

## 7. Therapeutic Potential of c-Cbl

The quintessential role of the ubiquitination-proteasomal system in fundamental cell processes has attracted intense attention of the pharmaceutical industry. In fact, this system is already a validated therapeutic target for several diseases including cancer and is explored in other diseases such as heart conditions, etc. [98,99].

**Figure 3:**
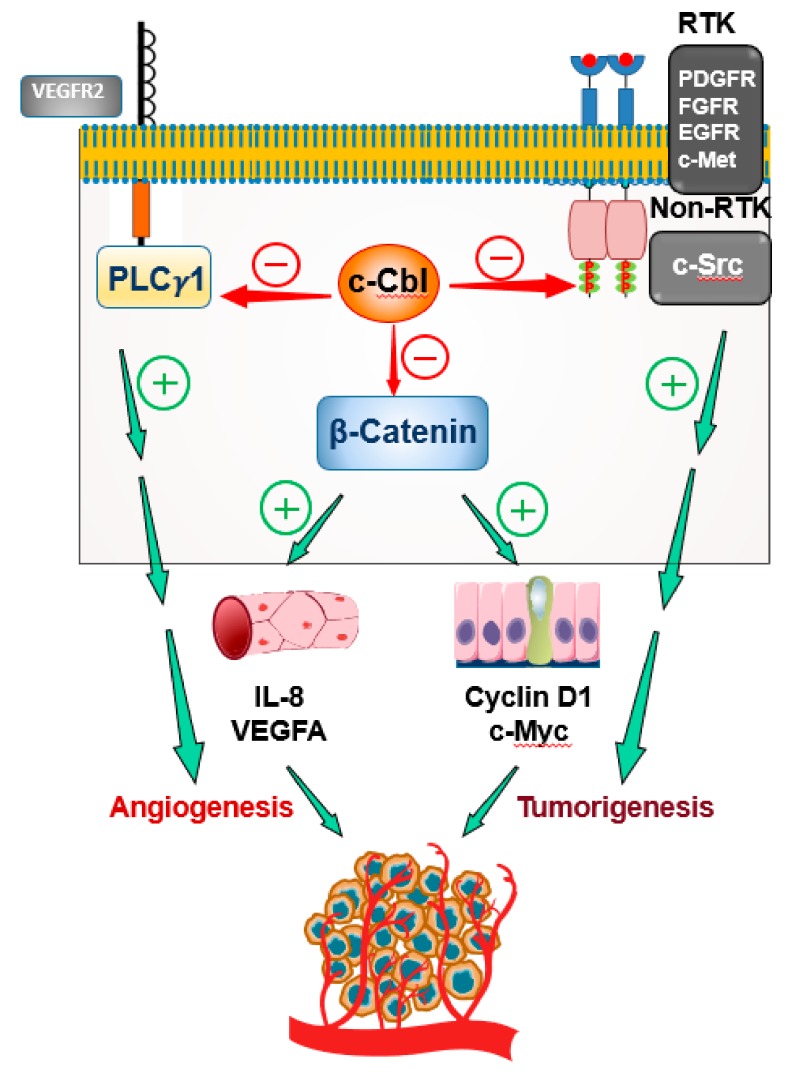
c-Cbl is an attractive therapeutic target for angiogenesis and tumorigenesis. In the endothelial cells of vessels supplying the tumor, Wnt activation results in secretion of pro-angiogenic cytokines IL-8 and VEGF-A that augment tumor-induced angiogenesis. In colon cancer epithelial cells, inactivating mutations in APC tumor suppressor stabilizes β-Catenin and allows its nuclear translocation. This event results in the transcription of pro-proliferative and pro-oncogenic genes such as Cylin D1 and c-Myc to augment oncogenic activity. c-Cbl, by ubiquitinating nuclear β-Catenin in endothelial cells and in colon cancer cells, suppresses the growth of blood vessles and tumors. In addition, c-Cbl suppresses these processes by downregulating other pathways such VEGF signaling (PLCγ1) and mitogenic signaling (RTK and non-RTKs).

Currently, the inhibitors of ubiquitin-proteasome are the first line of therapy for paraproteinemias such as multiple myeloma [100]. Several preclinical studies and phase I clinical trials are underway for a range of compounds for variety of indications including liquid and solid cancers [98]. The inhibition of the ubiquitination-proteasomal system exerts a wide variety of effects such as cell death from the accumulation of ubiquitinated immunoglobin protein, induction of senescence, and anti-proliferative and immunomodulatory effects. While these global inhibitors of the ubiquitination-proteasomal system have paved the way for developing viable drug targets, targeting of E3 ubiquitin ligase remains an attractive proposal to drive specificity while minimizing the off-target effects.

c-Cbl represents an attractive target from several perspectives. For example, in the area of cancer, c-Cbl suppresses both tumorigenesis and angiogenesis (Figure 3). In colorectal cancer, c-Cbl suppresses the growth of colon cancer cells by downregulating Wnt signaling (a critical driver of colorectal cancer) and suppressing nuclear β–catenin ( a hallmark of Wnt activation) [47]. In fact, the expression of c-Cbl in colorectal tumors inversely correlated with the overall survival of patients [48]. c-Cbl also suppresses other mitogenic proteins such as c-MET, PDGFR and FGF that are upregulated in colorectal and other cancers [10,42,101]. At the same time, c-Cbl inhibits tumor-induced angiogenesis by targeting the Wnt/β–catenin pathway, VEGFR-2 signaling, Angiopoietin(1/2)-Tie2 axis, and other tyrosine kinases [25]. c-Cbl is highly expressed in immune cells such as T-lymphocytes and myeloid cells. In fact, a global c-Cbl knockout was characterized by immune cell proliferation and splenomegaly [102,103]. Recent explosion in the field of immune oncology has underscored the profound influence these cells and their receptors have on tumor growth [104,105]. Alteration of the tumor milieu by modulation of c-Cbl can exert an additional benefit, this area remains to be explored.

There are potential solutions regarding the concerns around targeting c-Cbl. Diseases such as cancer will warrant activation of c-Cbl for its therapeutic value. In general, activation of a protein is considered challenging compared to inhibiting a protein. However, c-Cbl’s crystal structure provides an intriguing possibility of addressing this limitation [29]. Constitutively c-Cbl remains in an autoinhibited conformation. The activation of E3 ubiquitin ligase is impacted by phosphorylation of Tyr371. The phosphorylation status of Tyr371 is important for its ability to suppress Wnt signaling in cells and animals [48]. It is conceivable that targeting the kinases or phosphatases modulating the phosphorylation of this particular tyrosine residue can be exploited to drive therapeutic benefit. c-Cbl is ubiquitously expressed protein and its manipulation can have off-target effects. This limitation can be circumvented by technologies that precisely target cell-types or organs. Biomarkers accompanying a therapeutic strategy, helps to narrow the patient population to maximize the benefit and offset the off-target effects. In this case, targeting of c-Cbl can be linked to its activity in the organ of interest and the antibody-based detection of phosphorylation of Tyr371 can potentially serve as a marker of c-Cbl activity. Development of such resources will enrich the understanding of the pattern of activity of c-Cbl in relationship to development of a disease model. Eventually, such know-how will drive the therapeutic potential of c-Cbl.

## 8. Conclusions

c-Cbl as a deterrent of angiogenesis is both interesting and highly compelling. Regulation of key angiogenic factors by c-Cbl underscores its importance in this fundamental biological process. Independent of its suppressive effect on angiogenesis, c-Cbl’s ability to regulate tumor growth and potentially modulate immune cells to orchestrate potent growth inhibitory effects on tumors, makes it an appealing target for cancer therapeutics. Further work in some of the underexplored aspects of c-Cbl biology such as biomarkers of its activity and it’s role in other models of angiogenesis (such as hind limb ischemia and a therapeutic strategy to specifically target it), will allow realization of its true potential as a critical regulating node in the fabric of several disease processes.
